# Subtype-Specific Breast Cancer Incidence Rates in Black versus White Men in the United States

**DOI:** 10.1093/jncics/pkz091

**Published:** 2019-12-12

**Authors:** Hyuna Sung, Carol DeSantis, Ahmedin Jemal

**Affiliations:** Surveillance and Health Services Research Program, American Cancer Society, Atlanta, GA

## Abstract

Compared with white women, black women have higher incidence rates for triple-negative breast cancer but lower rates for hormone receptor (HR)–positive cancers in the United States. Whether similar racial difference occurs in male breast cancer is unclear. We examined racial differences in incidence rates of breast cancer subtypes defined by HR and human epidermal growth factor receptor 2 (HER2) by sex using nationwide data from 2010 to 2016. Among men, rates were higher in blacks than whites for all subtypes, with the black-to-white incidence rate ratios of 1.41 (95% confidence interval [CI ]= 1.32 to 1.50) for HR+/HER-, 1.65 (95% CI = 1.40 to 1.93) for HR+/HER2+, 2.62 (95% CI = 1.48 to 4.43) for HR-/HER2+, and 2.27 (95% CI = 1.67 to 3.03) for triple-negative subtype. Conversely, among women, rates in blacks were 21% lower for HR+/HER2- and comparable for HR+/HER2+ but 29% and 93% higher for HR-/HER2+ and triple-negative subtypes, respectively. Future studies are needed to identify contributing factors to the dissimilar racial patterns in breast cancer subtype incidence between men and women.

In the United States, incidence rates of breast cancer in men are higher in blacks than in whites [1,2]. In contrast, among women, breast cancer incidence rates remain slightly higher in whites than in blacks [3]. However, there are considerable racial differences in breast cancer rates by subtype, with black women approximately having two fold higher incidence rates of triple-negative breast cancer but lower rates of hormone receptor (HR)–positive cancers [4]. This difference has significant implications for etiological heterogeneity, patient management, and racial disparity in survival [5]. However, it is unknown whether similar subtype-specific differences in breast cancer incidence rates occur between black men and white men. Herein, we examined subtype-specific breast cancer incidence rates in black and white men in the United States using a contemporary nationwide database.

We obtained data for invasive breast cancers diagnosed in men (≥20 years) from 2010 to 2016 and reported to National Program of Cancer Registries and the Surveillance, Epidemiology, and End Results Program [6], covering the entire US population. Race/ethnicity was classified into white non-Hispanic (whites) and black non-Hispanic (blacks). Breast cancer subtypes were classified based on joint hormone receptor (HR, estrogen receptor, and progesterone receptor) and human epidermal growth factor receptor 2 (HER2) status [5]. We calculated average annual age-standardized incidence rates between 2010 and 2016 by race and subtype using the 2000 US standard population. Differences in subtype-specific rates between black and white men were expressed as incidence rate ratios (IRRs) and 95% confidence intervals (CI) using the rate in whites as the reference. We similarly examined incidence patterns in black and white women. Analyses were conducted using SEER*Stat software version 8.3.5. All tests were two-sided and considered significant at *P *less than* *.05.

There were 11 990 (84.0% white, 16.0% black) male and 1 267 147 (86.3% white, 13.7% black) female breast cancer cases. Table 1 shows overall and subtype-specific incidence rates of breast cancer in men and women by race, and Figure 1 shows corresponding black-to-white IRRs (95% CI). Among men, overall breast cancer incidence rates were 52% higher in black than in white men (2.75 vs 1.81 per 100 000 men), whereas among women, rates were 2% lower in blacks than in whites (177.00 vs 181.53 per 100 000 women). The higher incidence rates in black men than white men involved all subtypes, with IRRs of 1.41 (95% CI = 1.32 to 1.50) for HR+/HER2-, 1.65 (95% CI = 1.40 to 1.93) for HR+/HER2+, 2.62 (95% CI = 1.48 to 4.43) for HR-/HER2+, and 2.27 (95% CI = 1.67 to 3.03) for triple-negative subtypes. In contrast, among women, rates in blacks compared with whites were 21% lower for HR+/HER2- subtype and comparable for HR+/HER2+ subtype but 29% and 93% higher for HR-/HER2+ and triple-negative subtypes, respectively.

**Figure 1.**
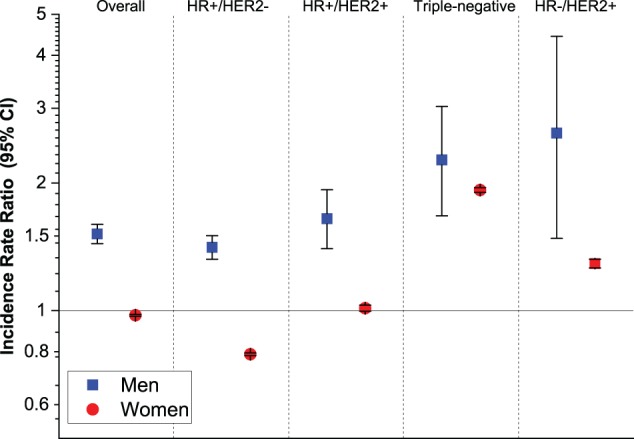
Black-to-white incidence rate ratios (95% CI) for breast cancer subtypes defined by joint HR/HER2 stratified by sex in the United States, 2010–2016. Incidence in non-Hispanic whites was assigned at relative risk of 1 and used as the reference. **Error bars** represent 95% CIs, which were estimated based on Tiwari method using SEER*Stat software (seer.cancer.gov/seerstat) version 8.3.5. CI = confidence interval; HER2 = human epidermal growth factor receptor 2; HR = hormone receptor.

**Table 1.** Age-standardized incidence rates per 100 000 person-years (20 years or older) for invasive breast cancer overall and subtypes by sex and race/ethnicity, and rate ratios (95% CI) relative to non-Hispanic whites in the United States, 2010–2016*

Breast cancer subtype and race/ethnicity	Men					Women				
	No. of cases	Rate	(SE)	Rate ratio (95% CI)	*P*_rate ratio_	No. of cases	Rate	(SE)	Rate ratio (95% CI)	*P*_rate ratio_
Overall	11 990					1 276 147				
White non-Hispanic	10 069	1.81	(0.019)	1.00 (Referent)		1 101 861	181.5	(0.180)	1.00 (Referent)	
Black non-Hispanic	1921	2.75	(0.067)	1.52 (1.44 to 1.60)	<.0001	174 286	177.0	(0.433)	0.98 (0.97 to 0.98)	<.0001
HR+/HER2-	8407					824 608			
White non-Hispanic	7141	1.28	(0.016)	1.00 (Referent)		732 268	119.4	(0.145)	1.00 (Referent)	
Black non-Hispanic	1266	1.81	(0.054)	1.41 (1.32 to 1.50)	<.0001	92 340	94.2	(0.316)	0.79 (0.78 to 0.79)	<.0001
HR+/HER2+	1199					116 685				
White non-Hispanic	989	0.18	(0.006)	1.00 (Referent)		99 164	17.4	(0.058)	1.00 (Referent)	
Black non-Hispanic	210	0.29	(0.021)	1.65 (1.40 to 1.93)	<.0001	17 521	17.6	(0.136)	1.01 (1.00 to 1.03)	.11
Triple negative	287					134364				
White non-Hispanic	220	0.04	(0.003)	1.00 (Referent)		101 006	17.4	(0.057)	1.00 (Referent)	
Black non-Hispanic	67	0.09	(0.012)	2.27 (1.67 to 3.03)	<.0001	33 358	33.5	(0.187)	1.93 (1.90 to 1.95)	<.0001
HR-/HER2+	84					48 384				
White non-Hispanic	62	0.01	(0.002)	1.00 (Referent)		39 554	6.8	(0.036)	1.00 (Referent)	
Black non-Hispanic	22	0.03	(0.007)	2.62 (1.48 to 4.43)	.001	8830	8.8	(0.095)	1.29 (1.26 to 1.32)	<.0001
Unknown	2013					152 106				
White non-Hispanic	1657	0.30	(0.008)	1.00 (Referent)		129 869	20.6	(0.060)	1.00 (Referent)	
Black non-Hispanic	356	0.53	(0.030)	1.75 (1.55 to 1.98)	<.0001	22 237	22.9	(0.157)	1.11 (1.10 to 1.13)	<.0001

* The analysis included data from 50 states and the District of Columbia. The year 2010 is the first year when HER2 was routinely collected. HR status was defined as HR+ if ER or PR was positive or borderline and as HR- if both ER and PR were negative. For HER2, borderline was considered unknown [5]. CIs for rate ratios were calculated based on Tiwari method using SEER*Stat software (seer.cancer.gov/seerstat) version 8.3.5. CI = confidence interval; ER = estrogen receptor; HER2 = human epidermal growth factor receptor 2; HR = hormone receptor; PR = progesterone receptor; SE = standard error.

Using nationwide US cancer registry data, we found that black men had considerably higher incidence rates for all breast cancer subtypes defined by HR/HER2 status compared with white men. Reasons for the elevated risk of breast cancer in black men are largely unknown but may involve a multitude of risk factors including genetic and nongenetic factors. Well-known risk factors for male breast cancer include family history of breast and/or ovarian cancers, pathogenic mutations in *BRCA2*, radiation exposure, and conditions that alter hormonal balance such as Klinefelter syndrome and gynecomastia, and potentially obesity and diabetes [7–9]. Several mutations in moderate-penetrance genes, including *CHEK2* and *PALB2*, and a few genetic loci possessing common variants have been also identified in relation to male breast cancer [9–12]. Moreover, a higher level of prediagnostic estradiol was found to be associated with increased risk of male breast cancer after controlling for body mass index [13], suggesting a presence of estrogen-mediated carcinogenesis in male breast cancer. However, whether associations of these risk factors vary by tumor subtypes remains unknown and should be considered in future etiologic studies.

Our novel finding is that incidence rates for HR+ breast cancers are considerably higher in black men than white men, in stark contrast to lower incidence rates in black women than white women. Furthermore, this higher risk of HR+ cancers among black men than white men persisted across all age groups (Supplementary Figure 1, available online). Although racial differences in the prevalence of mammography [14] and menopausal hormone supplements [15] are thought to have contributed to the historically higher incidence rate of HR+ cancers in white women, these are not applicable to etiology of breast cancer in men.

A strength of our study is the use of nationwide data to provide the first report on differences in subtype-specific breast cancer incidence rates between black men and white men. The primary limitation of our study is unknown subtype information (17% in male and 12% in female cases). However, this is unlikely to affect the interpretation of our findings given that black men also had higher incidence rates for the subtype-unknown group (Supplementary Figure 2, available online). Black–white patterns in subtype-specific breast cancer incidence rates differ between men and women, especially for HR+ disease, which may have implications for breast cancer etiology. Future studies should identify factors contributing to these patterns to further inform prevention strategies.

## Funding

This work was supported by the Intramural Research Department of the American Cancer Society.

## Notes

Affiliation of authors: Surveillance and Health Services Research Program, American Cancer Society, Atlanta, GA (HS, CD, AJ).

All authors are employed by the American Cancer Society, which receives grants from private and corporate foundations, including foundations associated with companies in the health sector for research outside the submitted work. The authors are not funded by or key personnel for any of these grants, and their salary is solely funded through the American Cancer Society.

The funder had no role in the design and conduct of the study; collection, management, analysis, and interpretation of the data; preparation, review, or approval of the manuscript; and decision to submit the manuscript for publication.

The authors gratefully acknowledge all cancer registries and their staff for their hard work and diligence in collecting cancer information, without which this research could not have been done.

## Supplementary Material

pkz091_Supplementary_Data
