# Supplementary material for: Subtype-Specific Breast Cancer Incidence Rates in Black versus White Men in the United States
Source: JNCI Cancer Spectr. 2019 Dec 12;4(1):pkz091. doi: 10.1093/jncics/pkz091 (PMC7050146; doi:10.1093/jncics/pkz091)
Supplement: pkz091_Supplementary_Data [file pkz091_supplementary_data.pdf]

Supplementary Figure 1. Age-specific breast cancer incidence rates by race/ethnicity according to HR/HER2 subtype in the US, 2010-2016

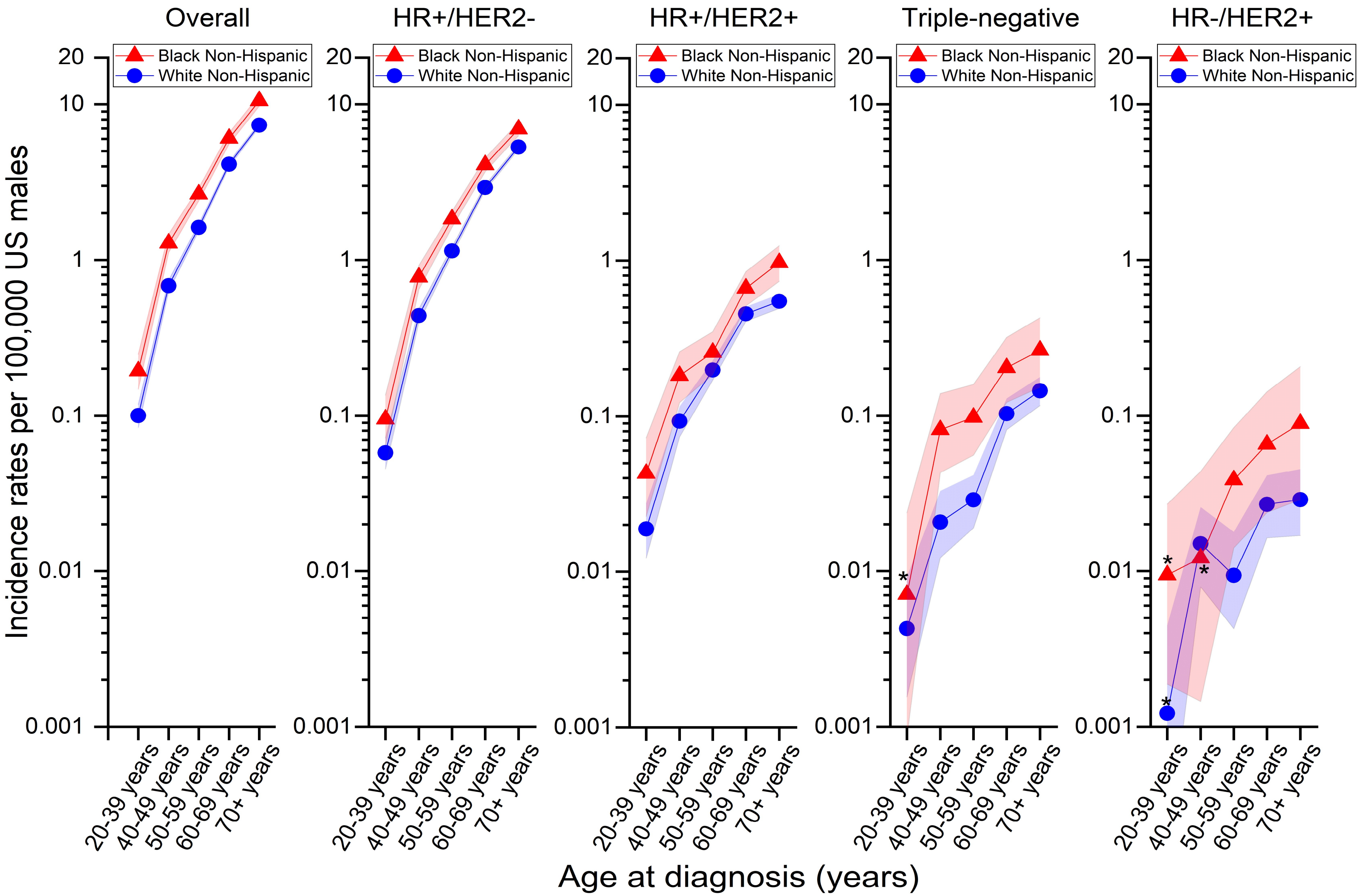

Abbreviations: CI, confidence interval; HER2, human epidermal growth factor receptor 2; HR, hormone receptor

NOTE *Incidence rates were calculated based on case number less than 5 which needs cautious interpretation.

Supplementary Figure 2. Age-specific incidence rates of unknown HR/HER2 breast cancer by race/ethnicity in the US, 2010-2016

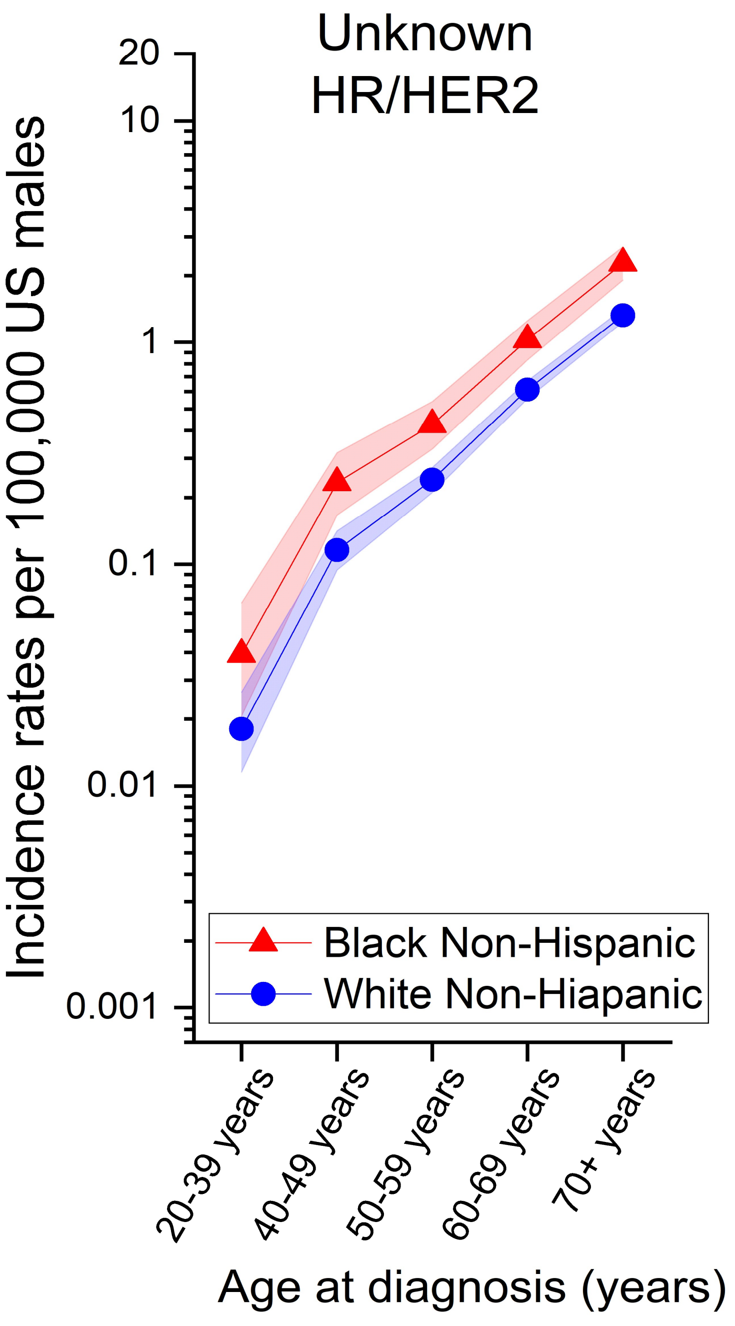

Abbreviations: CI, confidence interval; HER2, human epidermal growth factor receptor 2; HR, hormone receptor
